# The Ameliorating Effect of Steamed and Fermented *Codonopsis lanceolata* on Scopolamine-Induced Memory Impairment in Mice

**DOI:** 10.1155/2013/464576

**Published:** 2013-07-14

**Authors:** Jin Bae Weon, Bo-Ra Yun, Jiwoo Lee, Min Rye Eom, Ji Seon Kim, Hyeon Yong Lee, Dong-Sik Park, Hee-Chul Chung, Jae Youn Chung, Choong Je Ma

**Affiliations:** ^1^Division of Bioscience and Biotechnology, Department of Biomaterials Engineering, Kangwon National University, Chuncheon 200-701, Republic of Korea; ^2^Department of Teaics, Seowon University, Cheongju 361-742, Republic of Korea; ^3^Functional Food & Nutrition Division, Department of Agrofood Resources, Suwon 441-853, Republic of Korea; ^4^Newtree CO, LTD. 11F Tech Center, SKnTechno Park 190-1, Seongnam 462-120, Republic of Korea; ^5^Research Institute of Biotechnology, Kangwon National University, Chuncheon 200-701, Republic of Korea

## Abstract

*Codonopsis lanceolata* (Campanulaceae) have been traditionally used to treat lung inflammatory diseases, such as asthma, tonsillitis, and pharyngitis. The present study was performed to evaluate the cognitive-enhancing effects of steamed and fermented *C. lanceolata* in scopolamine-induced memory impairments in mice. Cognitive abilities were determined by the Morris water maze and passive avoidance tests. Mice orally received fermented *C. lanceolata* extract at doses of 100, 300, or 500 mg/kg body weight. Fermented *C. lanceolata* extract (500 mg/kg body weight, p.o.) significantly shortened the escape latency times that were increased by scopolamine on the 4th day of trial sessions in the Morris water maze task. In addition, it exerted longer step-through latency times than those of the scopolamine-treated group in the passive avoidance test. Furthermore, the neuroprotective effects of fermented *C. lanceolata* extract on glutamate-induced neurocytotoxicity were investigated in HT22 cells. Fermented *C. lanceolata* extract showed a relative protection ratio of 59.62% at 500 **μ**g/mL. In conclusion, fermented *C. lanceolata* extract ameliorated scopolamine-induced memory impairments, exerted neuroprotective effects, and improved activity compared to that found with original *C. lanceolata*. Further study will be required to investigate the mechanisms underlying this cognitive-enhancing activity.

## 1. Introduction

Alzheimer's disease (AD) is the most common form of dementia. AD is associated with neurodegeneration that is characterized by the accumulation of amyloid-*β*-containing plaques and neurofibrillary tangles that are composed of hyperphosphorylated tau [1–4]. A decrease in cholinergic function in the central nervous system is also associated with a decline in cognitive function and memory loss [5, 6]. Therefore, cholinesterase inhibitors are designed to protect the cholinergic system, which is essential for memory and learning. The main type of medication used in the treatment of AD is cholinesterase inhibitors, such as donepezil, galantamine, and tacrine [7, 8]. These medicines have several side effects, such as pain, nausea, and vomiting. The efficiency of treatments with medicinal plants, such as *Gingko biloba*, *Salvia officinalis* (sage), *Melissa officinalis* (balm), and *Papaver somniferum* (opium poppy), has been reported with less side effects [9].

*Codonopsis lanceolata *(*C*.* lanceolata*), which is a herb of the Campanulaceae family, have been used as a treatment for hypertension and several lung inflammatory diseases, such as asthma, tonsillitis, and pharyngitis, in East Asia for thousands of years. *C. lanceolata* are composed of various compounds, including saponins, alkaloids, tannins, steroids, and polysaccharides [10, 11]. A previous report has indicated that *C. lanceolata* show antilipogenic and anti-inflammatory effects in mice with alcohol-induced fatty liver [12]. In addition, it inhibits the production of tumor necrosis factor-*α* and nitric oxide, the expression of interleukin-3 and interleukin-6, and lipopolysaccharide-mediated phagocytic uptake in RAW 264.7 cells (regulatory effects of *C*.* lanceolata* on macrophage-mediated immune responses) [13, 14]. Scopolamine, which is a muscarinic antagonist, is an anticholinergic drug that causes memory impairments in animals and humans. Thus, scopolamine treatment represents a satisfactory model of learning and memory deficits, and it can be used to screen drugs for potential therapeutic usefulness [15]. We evaluated the cognitive-enhancing effects of fermented *C. lanceolata* on scopolamine-induced memory deficits in mice and its neuroprotective effects on glutamate-induced neurotoxicity in the mouse hippocampal HT22 cell. In addition, the effects of the *C. lanceolata* extract were compared to those of the original *C. lanceolata* extract (not fermented).

## 2. Materials and Methods

### 2.1. Plant Materials

The roots of *C. lanceolata* were collected from Hoengseong, Gangwon, Republic of Korea. *C. lanceolata* were washed with tap water and dried at 20–30°C for 2 days. Dried *C. lanceolata* were steamed with a steam device (Dechang Stainless, Seoul, Republic of Korea) at 90°C for 12 h and then dried for an additional 12 h. The above process was repeated 5 times.

### 2.2. Fermentation and Extraction

*Bifidobacterium longum* (KACC 20587), *Lactobacillus acidophilus* (KACC 12419), and *Leuconostoc mesenteroides* (KACC 12312) were obtained from the Korean Agricultural Culture Collection (Suwon, Republic of Korea). The steamed *C. lanceolata* were mixed in distilled water that was 8 times the weight of the herbs and aseptically inoculated with approximately 10^6^ CFU/g of *B. longum*, *L. acidophilus*, and *L. mesenteroides*. The inoculated *C. lanceolata* were fermented for 48 h at 37°C. Then, the cultures were harvested by spinning at 5,000 rpm for 10 min at 4°C. Fermented *C. lanceolata* were extracted in 70% ethanol (100 kg/10,000 L) for 24 h of reflux extraction at 100°C. After evaporation, it was freeze-dried in order to obtain the fermented *C. lanceolata*.

### 2.3. Animals

ICR mice weighing between 25 and 30 g (males, 3 weeks of age) were used in the present study (Dae Han Biolink Co., Ltd., Eumseong, Republic of Korea). The mice were housed 7 per cage in a room under a 12/12 h light-dark cycle and controlled temperature (20 ± 3°C) with free access to commercial pellet feed and water *ad libitum*. The mice were used after a 1-week adaptation period. All animal experimental procedures in this study were conducted according to the guidelines of the Kangwon National University IACUC.

### 2.4. Morris Water Maze Test

The water maze test was performed according to the previously described Morris methods with some modifications in order to assess spatial learning and memory in mice [16]. The water maze consisted of a circular pool (90 cm in diameter and 40 cm in height) that was filled to a depth height of 30 cm with water in which 500 mL of white milk had been mixed and maintained at 20 ± 1°C. The maze was divided into 4 equal quadrants. The starting points of the test were marked on the outside of the pool as north (N), south (S), east (E), and west (W). A white escape platform (10 cm in diameter and 29 cm high) was located in the center of 1 quadrant of water maze and submerged 1 cm below the water surface so that it was invisible at water level. All swimming behaviors of the mice were monitored and analyzed by a Smart (version 2.5.21) video-tracking system. The escape latency, which was the time required to locate the platform, was used as a measure of the development of spatial memory. On the first day, mice were given 60 s to swim in the absence of the platform the day prior to the test. The mice received 2 trial sessions per day for 4 consecutive days with an intertrial interval of 20 min. The location of the platform was unchanged between trials 1 and 2 during the test period, but the starting point was changed each day. Once the mouse reached the platform, it was allowed 10 s to stay on the platform. If the mouse did not locate the platform within 120 s, the trial was stopped, the mouse was placed on the platform for 10 s, and the escape latency was recorded as 120 s. A probe trial was performed for a time period of 60 s without the platform on the last day in order to investigate the time spent in the target quadrant. The time spent in the correct quadrant was recorded as a measure of spatial memory. Oral doses of 0.5% carboxymethylcellulose (CMC; control group), *C. lanceolata* (100, 300, or 500 mg/kg, dissolved in CMC), or donepezil (1 mg/kg), which was used as a positive control, were administered daily 90 min before treatment with scopolamine. The control group was subcutaneously administered normal saline, and all of the other groups (donepezil and sample groups) were subcutaneously given scopolamine (1 mg/kg dissolved in saline) in order to induce amnesia. The first test trial was performed 30 min after scopolamine treatment.

### 2.5. Passive Avoidance Test

The passive avoidance apparatus (Gemini system, San Francisco, CA, USA) consisted of 2 compartments, which were equally sized light and dark compartments (17 cm × 12 cm × 10 cm) and which were equipped with an electric grid floor. A guillotine door was placed in the center of the partition between the 2 compartments. A training trial was performed on the first day; the mice were initially placed in the light compartment. The door between the 2 compartments was opened 20 s later. When the mice moved into the dark compartment, the guillotine door closed automatically, and an electric foot-shock (0.1 mA/10 g body weight) of a 2 s duration was delivered through the grid floor. During each trial, the time taken to enter the dark compartment was recorded as the training latency. Mice that did not enter the dark compartment within 180 s were excluded from the experiment. Doses of 0.5% CMC (control group), *C. lanceolata* (100, 300, or 500 mg/kg body weight), or donepezil (1 mg/kg), which was used as a positive control, were orally administered 90 min before treatment with scopolamine. Amnesia was induced by scopolamine (1 mg/kg body weight), which was given subcutaneously, and the training trial was performed 30 min after treatment with scopolamine. Twenty-four hours after the training trial, the mice were again placed in the light compartment, and the latency time was measured.

### 2.6. Cell Viability

Cell viability was determined by an MTT assay. Mouse hippocampal HT22 cells were provided by Seoul National University, Republic of Korea. HT22 cells were seeded at a density of 6.7 × 10^4^ cells/well (48-well plate) in Dulbecco's Modified Eagle's Medium that was supplemented with 10% (v/v) fetal bovine serum, 1% penicillin/streptomycin, NaHCO_3_ (2 mg/mL), and 15 mM HEPES and then incubated at 37°C at 5% CO_2_ for 24 h. After incubation of the HT22 cells with glutamate in the presence or absence of 10, 100, or 500 *μ*g/mL of *C. lanceolata* for 24 h, MTT solution (1 mg/mL) was added to each well and incubated for 3 h. Dimethyl sulfoxide was added, and the optical density of the solubilized formazan product in each well was measured at 570 nm with an enzyme-linked immunosorbent assay reader.

### 2.7. High-Performance Liquid Chromatography (HPLC) Analysis of Phenolic Compounds

 The contents of phenolic compounds, including gallic acid, 4-hydroxybenzoic acid, caffeic acid, vanillic acid, 4-coumaric acid, trans-ferulic acid, and caffeine, in fermented *C. lanceolata* were analyzed with an HPLC system (Agilent 1260 series, Agilent Technologies, Inc., Santa Clara, CA, USA) that was equipped with a diode array UV/VIS detector. Separation was performed on a ZORBAX Eclipse XDB-C_18_ (250 × 4.60 mm i.d., 5 *μ*m), and the column temperature was maintained at 35°C. The mobile phase consisted of 10% acetonitrile with 0.1% formic acid (A) and 0.1% formic acid in 40% acetonitrile and 40% methanol (B) that was applied at a flow rate of 1 mL/min. A gradient elution system of the mobile phase was used to achieve the analysis (0–15 min, 95% A; 15–23 min, 60% A; 23–33 min, 60% A; 33–42 min, 0% A; 42–45 min, 95% A; and 45–50 min, 95% A). The detection UV wavelength was set between 190 nm and 400 nm for a UV maximum wavelength of 7 compounds, and the UV wavelength was set at 280 nm. The sample injection volume was 20 *μ*L.

### 2.8. Statistical Analysis

All results were expressed as means ± standard error of the mean. The escape latency in the Morris water maze test was analyzed by two-way analysis of variance (ANOVA). The probe trial test data in the Morris water maze test value was analyzed by one-way ANOVA followed by the Student-Newman-Keuls test. For the passive avoidance test value was analyzed by a Kruskal-Wallis nonparametric ANOVA test. If the results were significant, the significant differences were tested with Tukey's post hoc tests. Statistical significance was set at *P* < 0.05,  *P* < 0.01, and *P* < 0.001.

## 3. Results

### 3.1. Morris Water Maze Test

**Figure 1:**
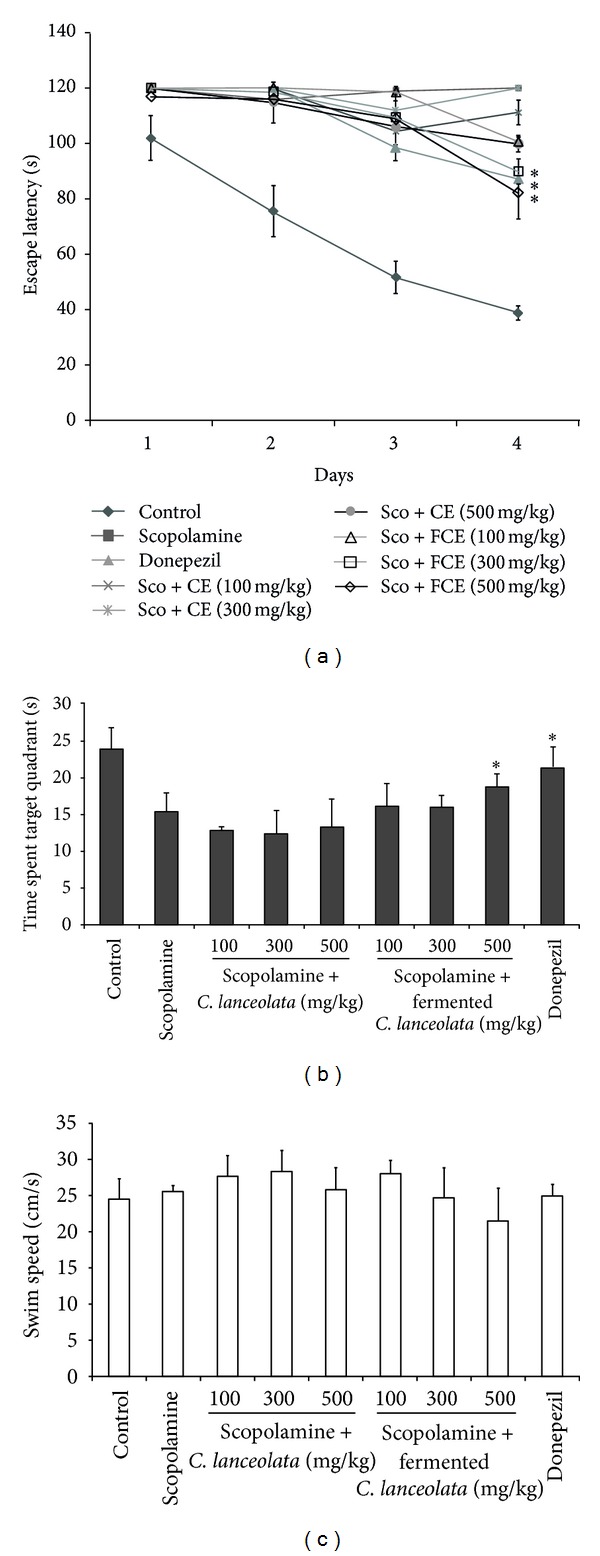
Effects of fermented *Codonopsis lanceolata* on escape latencies in the Morris water maze test in mice that were treated with control, scopolamine, donepezil, scopolamine (Sco) + fermented *C. lanceolata* extract (FCE) (100, 300, or 500 mg/kg), and scopolamine (Sco) +* C. lanceolata* extract (CE) (100, 300, or 500 mg/kg). (a) The escape latencies in the Morris water maze during the 4 days. The values shown are the mean escape latency ± standard deviation (SD; *n* = 7).   *P < 0.05, **P < 0.01, and  ***P < 0.001 compared to the scopolamine group. (b) The time spent in the quadrant where the platform was once placed in the probe test. The results are expressed as mean ± SD (*n* = 7).   *P < 0.05, **P < 0.01, and ***P < 0.001 compared to the control group. (c) Mean swimming speed of mice during 4 days in Morris water maze test. The data represent means ± SD. (*n* = 7)  *P < 0.05, **P < 0.01, and ***P < 0.001 compared with control group.

We determined the spatial learning and memory abilities of mice with the Morris water maze test, and the average of the 2-trial sessions for each day was evaluated (Figure 1). The mean latency of the control group decreased progressively during the 4 training days. Donepezil reduced the escape latencies after day 2, and they were significantly shortened on the last day. The mean escape latency of *C. lanceolata* (500 mg/kg) decreased after day 2, and the mean escapes latencies were significantly reduced on the 4th day of the test. In the Morris water maze test, the effects on the fermented *C. lanceolata* extract-treated group were significant for treatment (*F* (5,192) = 1.65, *P* < 0.001), days (*F* (3,192) = 2.07, *P* < 0.01), and for the interaction between treatment and day (*F* (15,192) = 1.31, *P* = 0.99). Furthermore, the fermented *C. lanceolata* extract-treated group had decreased latency times compared to those of the original *C. lanceolata*-treated group. In the probe trial on the last day, the swimming time within the target quadrant for the fermented *C. lanceolata*-treated group was dose dependently increased compared to that of the scopolamine-treated group. However, significant differences between groups in swimming speed during 4-day trials were not observed (Figure 1(c)).

### 3.2. Passive Avoidance Test

**Figure 2:**
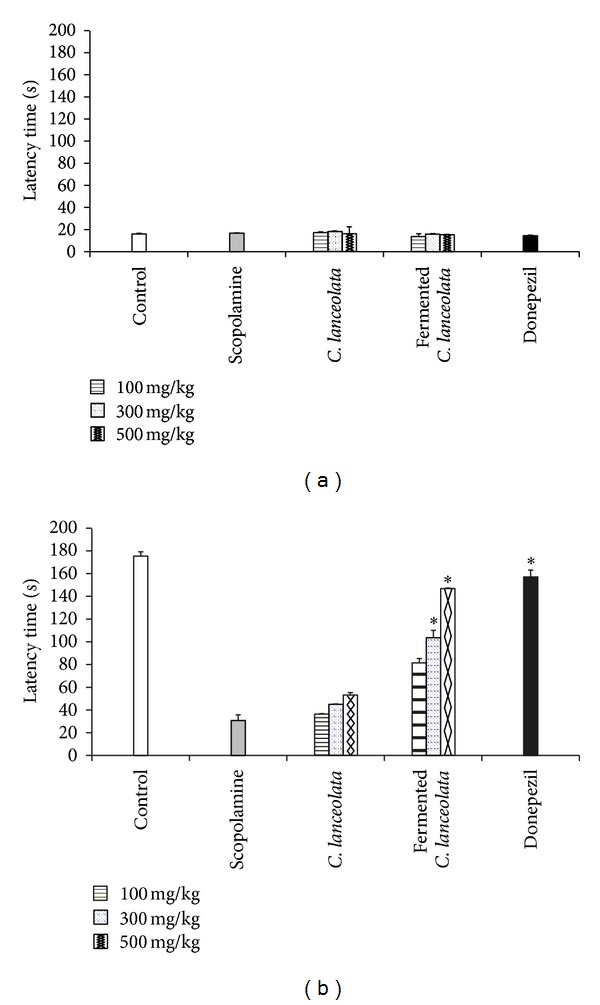
Effects of fermented *C. lanceolata* on scopolamine-induced memory impairments in mice in the passive avoidance test: (a) training trial, (b) test trial. The values shown are the mean latency time (s) ± SD (*n* = 7).   *P < 0.05, **P < 0.01, and ***P < 0.001 compared to the scopolamine group.

We investigated memory abilities with the use of the passive avoidance test as a behavioral task. The step-through latency time during the training trial was not affected by scopolamine, donepezil, and *C. lanceolata* extract sample (Figure 2(a)). The scopolamine group exhibited a decreased step-through latency time. Donepezil treatment was effective. *C. lanceolata* extract treatment at a dose of 500 mg/kg significantly prolonged the latency time (Figure 2(b)). Compared to the original *C. lanceolata* extract, the fermented *C. lanceolata* extract resulted in improvements in the memory-enhancing effects.

### 3.3. Cell Viability

**Figure 3:**
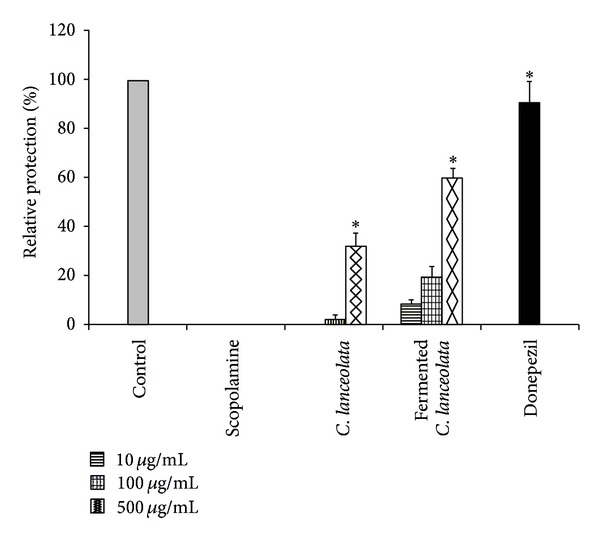
The neuroprotective effects of fermented *C. lanceolata* against glutamate-induced cytotoxicity in neuronal HT22 cells. Each bar represents the mean relative protection ± SD of 3 independent experiments. *P < 0.05, **P < 0.01, and ***P < 0.001 versus glutamate-injured cells.

 In order to evaluate the neuroprotective effects of fermented *C. lanceolata*, its protective effects on glutamate-induced cell death in HT22 cells were tested. Trolox, which is a well-known positive control against glutamate-induced cytotoxicity, exhibited neuroprotective activity at a concentration of 50 *µ*M. Fermented *C. lanceolata* were found to exert protective effects on HT22 cells that were treated with glutamate (relative protective ratio: 59.62% at 500 *µ*g/mL). As shown in Figure 3, fermented *C. lanceolata* attenuated the cytotoxicity that was induced by glutamate compared to that found with the original *C. lanceolata*.

### 3.4. HPLC Analysis of Phenolic Compounds

**Figure 4:**
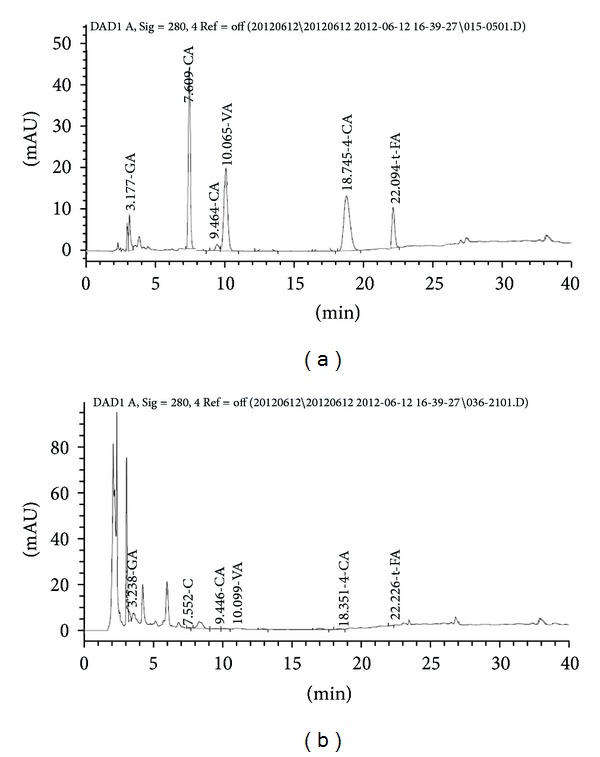
HPLC chromatogram of six phenolic standard compounds (a) and fermented *C. lanceolata *extract (b). GA: gallic acid; CA: caffeic acid; VA: vanillic acid; 4-CA: 4-coumaric acid; t-FA: trans-ferulic acid; C: caffeine.

The phenolic compounds in fermented *C. lanceolata* were measured with a HPLC-diode array detection (DAD) analysis (Figure 4). Phenolic compounds, including gallic acid, 4-hydroxybenzoic acid, caffeic acid, vanillic acid, 4-coumaric acid, trans-ferulic acid, and caffeine, were quantified. The contents of each of the respective phenolic compounds were calculated from the peak areas, and they were determined to be gallic acid (3,794 *µ*g/g), caffeic acid (90 *µ*g/g), vanillic acid (11 *µ*g/g), 4-coumaric acid (18 *µ*g/g), trans-ferulic acid (257 *µ*g/g), and caffeine (14 *µ*g/g). Gallic acid and trans-ferulic acid were the main phenolic acids (Table 1). Gallic acid was found to have the highest concentration, while 4-hydroxybenzoic acid was not detected.

## 4. Conclusions

In this study, we evaluated the cognitive-enhancing effects of fermented *C. lanceolata* for treating and preventing memory and learning deficits. We examined the effects of fermented *C. lanceolata* on scopolamine-induced memory impairments in the Morris water maze test and the passive avoidance test. Scopolamine is a nonselective muscarinic antagonist that blocks cholinergic neurotransmission without changing the acetylcholinesterase (AchE) concentration and impairs cognitive functions, including learning and memory (long-term and short-term memories) [17, 18]. Cognitive function impairments in the scopolamine-induced animal model have also been shown to be associated with increased oxidative stress within the brain [19]. Donepezil as AchE inhibitor has been used for AD patients. Donepezil reverses cognitive deficit by improving cholinergic activity. The Morris water maze test evaluates spatial reference memory and is dependent on the hippocampus. In the Morris water maze test, scopolamine increased the escape latency times over the 4 days. The administration of fermented *C. lanceolata* shortened the escape latency times between days 2 and 4. At the probe trial, fermented *C. lanceolata* increased the swimming time that was spent in the target quadrant. The passive avoidance test was used to evaluate the effects of drug affecting learning and memory. Fermented *C. lanceolata* ameliorated the reduced step-through latency time that was induced by scopolamine treatment. Fermentation significantly improved the cognitive-enhancing activities in the Morris water maze test and passive avoidance test compared to those of the original *C. lanceolata*. In addition, we found neuroprotective effects of fermented *C. lanceolata* on the cell death that was induced by glutamate in HT22 cells. Glutamate is an excitatory neurotransmitter in the central nervous system. At high concentrations, glutamate is involved in neuronal cell death by oxidative cell death [20, 21]. Oxidative stress is thought to contribute to the pathogenesis of neurodegenerative diseases. We investigated the contents of the phenolic compounds, including gallic acid, 4-hydroxybenzoic acid, caffeic acid, vanillic acid, 4-coumaric acid, trans-ferulic acid, and caffeine, in fermented *C. lanceolata* with an HPLC-DAD analysis. The content of gallic acid is higher than those of other compounds. Gallic acid is known to have AchE inhibitory activity and antioxidant effects [22, 23]. Ferulic acid also showed that antiamnesic effect against *β*-amyloid induced cognitive impairments [24]. The cognitive-enhancing and neuroprotective effects of fermented *C. lanceolata* are thought to be associated with the antioxidant activities, AchE inhibitory activity, and antiamnesic effect of gallic acid and ferulic acid. In a previous study, fermentation with lactic acid bacteria improved the bioactivity of a herb by converting glucoside to aglycon [25, 26]. Hence, we suggest that the antioxidant effects of *C. lanceolata* were enhanced through fermentation and that these effects may be involved in the improvements of amnesia observed in mice. In conclusion, the present study revealed that fermented C. *lanceolata* could reverse scopolamine-induced memory impairments in mice through the antioxidant and fermentation-enhanced effects of *C. lanceolata*. The results of this study suggest that fermented *C. lanceolata* could be useful as a therapy of AD. Further study will be required to identify the exact mechanisms of fermented *C. lanceolata* in the treatment of AD.

## Figures and Tables

**Table 1:** Contents of the phenolic compounds in fermented *C. lanceolata* from HPLC analysis.

Phenolic compounds	Contents (*μ*g/g)
Gallic acid	3,794 ± 10.25
Caffeic acid	90 ± 2.45
Vanillic acid	11 ± 1.65
4-Coumaric acid	18 ± 1.35
Trans-ferulic acid	257 ± 5.28
Caffeine	14 ± 1.25
